# Deep Learning for Image Analysis in the Diagnosis and Management of Esophageal Cancer

**DOI:** 10.3390/cancers16193285

**Published:** 2024-09-26

**Authors:** Charalampos Theocharopoulos, Spyridon Davakis, Dimitrios C. Ziogas, Achilleas Theocharopoulos, Dimitra Foteinou, Adam Mylonakis, Ioannis Katsaros, Helen Gogas, Alexandros Charalabopoulos

**Affiliations:** 1 Department of Surgery, Metaxa Cancer Hospital, 18537 Piraeus, Greece; 2 First Department of Surgery, School of Medicine, Laiko General Hospital, National and Kapodistrian University of Athens, 11527 Athens, Greece; spdavakis@gmail.com (S.D.); adam.mylonakis@gmail.com (A.M.); gikats.md@gmail.com (I.K.); acharalabopoulos@yahoo.com (A.C.); 3 First Department of Medicine, School of Medicine, Laiko General Hospital, National and Kapodistrian University of Athens, 11527 Athens, Greece; ziogasdc@gmail.com (D.C.Z.); foteinoudimitra@yahoo.com (D.F.); helgogas@gmail.com (H.G.); 4 Department of Electrical and Computer Engineering, National Technical University of Athens, 10682 Athens, Greece; achilleastheo13@gmail.com

**Keywords:** esophageal cancer, Barrett’s esophagus, deep learning, convolutional neural networks, machine learning, computer-aided diagnosis

## Abstract

**Simple Summary:**

The implementation of artificial intelligence in healthcare has gained significant momentum over the last decade, particularly in specialties where image analysis and object identification apply. In esophageal diseases, DL has been primarily employed for image analysis in upper digestive endoscopy and histopathological specimens. Recent efforts aim to expand DL utilization into further aspects of esophageal neoplasia, opening new frontiers in the management of esophageal cancer. This review aims to collectively summarize the published body of evidence on DL approaches for image analysis in the management of esophageal cancer.

**Abstract:**

Esophageal cancer has a dismal prognosis and necessitates a multimodal and multidisciplinary approach from diagnosis to treatment. High-definition white-light endoscopy and histopathological confirmation remain the gold standard for the definitive diagnosis of premalignant and malignant lesions. Artificial intelligence using deep learning (DL) methods for image analysis constitutes a promising adjunct for the clinical endoscopist that could effectively decrease BE overdiagnosis and unnecessary surveillance, while also assisting in the timely detection of dysplastic BE and esophageal cancer. A plethora of studies published during the last five years have consistently reported highly accurate DL algorithms with comparable or superior performance compared to endoscopists. Recent efforts aim to expand DL utilization into further aspects of esophageal neoplasia management including histologic diagnosis, segmentation of gross tumor volume, pretreatment prediction and post-treatment evaluation of patient response to systemic therapy and operative guidance during minimally invasive esophagectomy. Our manuscript serves as an introduction to the growing literature of DL applications for image analysis in the management of esophageal neoplasia, concisely presenting all currently published studies. We also aim to guide the clinician across basic functional principles, evaluation metrics and limitations of DL for image recognition to facilitate the comprehension and critical evaluation of the presented studies.

## 1. Introduction

The implementation of artificial intelligence (AI) in healthcare has been increasingly gaining momentum over the last decade, particularly in specialties where image analysis and object identification apply. According to a bibliometric analysis conducted in 2023, the growth rate of healthcare-related AI publications between 2000 and 2021 was 37.88%, while 88.88% of the total studies were published after 2012 [1]. The advent of AI applications in medicine largely stems from the success of deep learning (DL), a subfield of machine learning (ML), in image classification tasks [2]. Briefly, DL systems employ multilayered neural networks that, upon exposure to large sets of training data, extract image features based on which they classify data [3]. Medical imaging and endoscopy comprise the two fields where DL is primarily utilized [4,5]. In esophageal diseases, DL has been primarily employed for image recognition in upper digestive endoscopy and histopathological specimens. However, despite the growing number of studies evaluating DL models in endoscopy, only 28% of gastroenterologists and endoscopists have undergone formal training in AI [6] and only 24.6% of endoscopists were moderately or very familiar with AI [7], in surveys conducted in 2022 and 2023, respectively. Comparable results have been reported among surgeons [8] and radiation oncologists [9]. Although these percentages constitute a significant improvement compared to previous studies [10], the majority of clinicians do not possess a robust understanding of the technological functioning of AI and its applications in clinical medicine. In this manuscript, we provide an introductory discussion on AI and DL for image interpretation, we present commonly used evaluation metrics and their inherent limitations and concisely discuss the limitations of DL models to facilitate the comprehension and critical evaluation of original research articles. Table 1 provides a concise explanation of common evaluation metrics used in classification and segmentation tasks. Furthermore, we review the published body of data on the use of DL for the analysis of endoscopic, histopathologic, radiological and intraoperative images that are used in the diagnosis and management of esophageal neoplasia. Table 2 provides a summary of studies evaluating DL models for image analysis for the diagnosis of BE, neoplasia in BE and ESCC, Table 3 provides a summary of studies evaluating DL models for image analysis for the prediction of survival and response to systemic therapy and the evaluation of response after treatment, Table 4 provides a summary of studies evaluating DL models for image analysis for intraoperative surgical guidance and the delineation of target volume prior to radiotherapy.

### 1.1. Artificial Intelligence and the Recognition Problem

AI is a subfield of computer science that aims to investigate the appropriate computational models for computing cognitive functions, including recognition. Recognition refers to the ability of the human brain to process signals captured by senses and map them to a discrete, finite set of classes. These classes encode a meaningful interpretation of the external environment and allow humans to proceed to further action. It is interesting to note that this recognition problem, which in many cases is solved almost trivially by the human brain, appears to be very challenging, at least from a mathematical perspective. A recognition function is a mapping H : S → C, where S is the input space and C is the set of labels. A computational model $M\left(H\right)$ refers to a systematic method to compute H. By a simple overview of the above definition, a mathematically inclined reader would realize that the space containing all possible functions H, which is referred to as the “hypotheses space” in the literature, does not possess many of the desired properties that would facilitate the search of a particular h of H that best fits a particular recognition problem. The emerging question, then, is rather obvious: What kind of approach should someone adopt in designing that computational model M? An idea, which is ubiquitous in computation theory and expresses the fundamental concept of “Divide and Conquer”, is to decompose the function H into certain simpler subfunctions $H_{1},H_{2},\ldots ,H_{n}$, the synthesis of which produces H: $H=H_{n}(H_{n-1}\left(\ldots \left(H_{1}\left(s\right)\right)\right)$. The prevalent paradigm in applied pattern recognition indicates that H should be decomposed in a feature/representation extraction stage and a discriminative stage. One of the main reasons why designing H is hard is that, in most cases, the raw signal representation is not suitable for class recognition purposes. A feature extraction stage transforms the raw input into a lower-dimensional and more informative representation that can be used by a discriminator to decide the input’s class. In the discriminative stage, given a feature space X that is already derived by the previous stage of the model’s architecture, the discriminative subfunction is responsible for mapping feature representations to the discrete set of classes. A rather obvious, but powerful, way to do that is to discriminate the space X into regions of points, where each region is assigned to a certain class. Medical diagnosis, both in the context of identifying causes from symptoms and in recognizing structures in images, can be mathematically formulated as a recognition problem. Considering the first case, the input x to the system is a series of symptoms and the class c is the predicted disease. Throughout the years, many machine learning-based methods have been adopted to solve this problem, varying from Decision Trees to Bayesian Networks. The advantage of these models, which is particularly important in medical applications, is their transparency: in general, a human expert can follow, understand and therefore trust their inference process. On the other hand, these models are designed to handle low-dimensional inputs and require domain-specific knowledge, meaning that features (“symptoms”) are not extracted by the model itself, but they are instead suggested by medical experience based on their recorded relationship with a series of diseases. These inherent limitations make them unsuitable for high-dimensional image recognition tasks, where deep learning functions as the preferred approach.

### 1.2. Deep Learning for Medical Image Interpretation

DL is an approach to the design of the computational model M that is inspired by the corresponding human brain neural mechanisms [52]. Unlike human-engineered ML, DL involves the automatic extraction of input features necessary for pattern recognition. Neuroscience evidence indicates that a simplified model of the brain, at least concerning the recognition problem, would be that of a multilayered neural network; there, neurons, which perform some simple nonlinear transformation on their inputs, are arranged in layers and information flows across the network layers via synaptic signals [53]. The computational capacity of such a structure stems from the combined processing performed by the neurons, which, when organized in layers, are able to compute subfunctions such as feature extraction and discrimination. The functions $H_{f}\left(x;w_{f}\right)$ and $H_{d}\left(x;w_{d}\right)$, which correspond to the feature extraction and discriminative stage, respectively, are parametric. A key idea is that this set of internal parameters ($w_{f}$, $w_{d}$) could potentially be adjusted through a learning process, in such a way that the trained model would perform well on an arbitrary set of input data. Considering a supervised context, where a large enough labeled set of data are available, the “performance” of the model on that training set could serve as an estimate of its “performance” on an unknown dataset. The model’s performance is typically quantified via a loss function $L\left(x_{training};w_{d},w_{f}\right)\;$, where $x_{training}$ refers to the training samples, and thus an optimal performance would require the minimization of that function with respect to $w_{d},w_{f}$. The formulation of the learning process within the context of mathematical optimization provides us with a series of gradient-based methods to implement that process [52], all of which rely on the backpropagation algorithm [54] to compute the gradient $\frac{\partial \;L}{\partial \;w}$. That joint training process of both representation and discriminative stages is one of the key elements to the success of DL models, especially compared to conventional ML techniques, where the two stages were designed independently with poor results [55].

### 1.3. CNN Architecture

**Figure 1:**
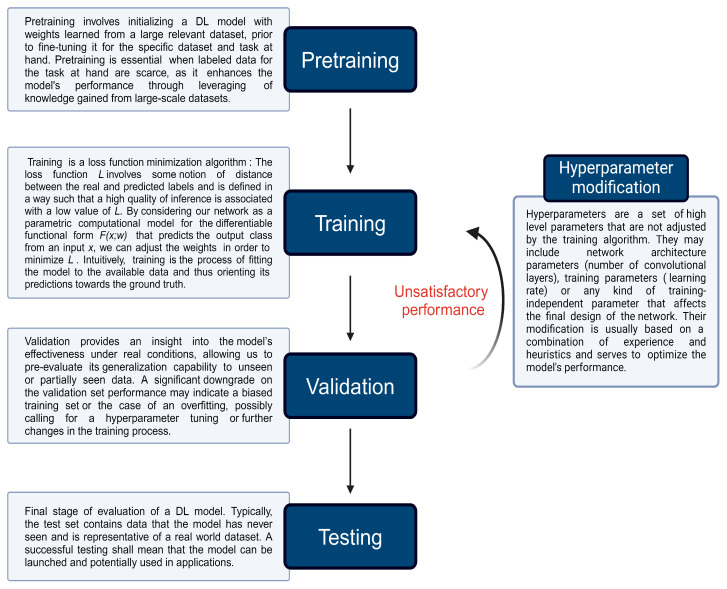
The core phases of building a DL model. Pretraining involves initializing the model parameters, using transfer learning from a pretrained model on a large dataset. Training adjusts these parameters using a labeled dataset, while validation fine-tunes the model by evaluating its performance on a separate validation set. The testing phase assesses the model’s generalizability on an unseen dataset to ensure it performs well in real-world scenarios.

**Figure 2:**
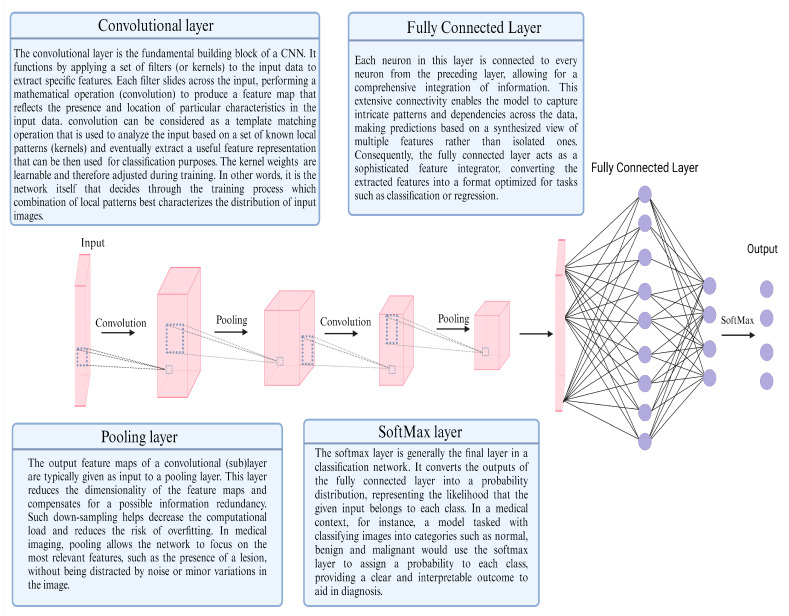
Schematic representation of a vanilla CNN architecture. A vanilla convolutional neural network (CNN) architecture comprises alternating convolutional and pooling layers that extract and down-sample features from input images. These layers are followed by fully connected layers that integrate the extracted features for classification, concluding with a SoftMax layer that provides the probability distribution across various classes.

A class of very popular and successful DL architectures for the specific task of image recognition and its variations, such as object detection, classification and semantic segmentation, are convolutional neural networks (CNNs) [56]. CNNs integrate the principle of a composite architecture, as exposed above, and their representation learning stage is believed to share common principles with the human visual recognition system [57]. The core of that stage, at least in the vanilla CNN, is a “convolution-pooling-activation” block, which is used N times in cascade and manages to automatically extract input characteristics to semantically meaningful high-level features and patterns. This allows for instantaneous output for real-time medical applications. The architecture consists of two main stages, a representation learning stage (R) and a discriminative stage (D) that are connected to each other in series. R is made up of a “Convolutional-pooling layer” repeated in cascade and D is essentially a multilayer perceptron, called a “Fully Connected layer” in the context of CNNs. The output vector of D is eventually passed through a SoftMax transformation, and the class is decided upon on the element of that vector that has the highest SoftMax score. In building a successful DL model, there exist four core phases: (1) pretraining, (2) training, (3) validation and (4) testing. Figure 1 discusses these four phases in a stepwise manner. Figure 2 presents a schematic representation of a vanilla CNN architecture. DL models can be implemented to assist in the interpretation of medical images through either computer-aided detection (CADe) or computer-aided diagnosis (CADx). CADe algorithms are constructed to detect pathology whereas CADx algorithms are developed to classify pathology.

### 1.4. Evaluation Metrics

Evaluation metrics comprise objective quantitative criteria that serve to assess the performance of DL models through the measurement of predictive ability and generalizability, while facilitating comparison between different models. The selection of evaluation metrics hinges on the data characteristics, type of model and desired outcomes. 

#### 1.4.1. Extension of Classification Metrics to Multiclass Problems

Accuracy can be directly applied to any classification task by simply considering the true and false predictions across all M classes. To extend recall, precision and F1 metrics to multiclass problems, a pair of aggregation techniques known as micro- and macro-averaging is employed.

Micro-averaging is targeted towards generalizing classification metrics for multiclass problems, in a way that each prediction is given an equal weight. As a result, the metric value is mainly determined by the model’s performance on majority classes, a feature that, depending on the application’s nature, may be desired or not: if all classes are of equal importance and we are interested in the large-scale performance of the model, then we are indeed seeking a metric able to capture a possible imbalance in the classes’ population. On the other hand, a micro-average metric carries little information about minority classes, thus being unsuitable for applications where these are of highest importance. Micro-average precision, recall and F1 scores are defined as the following:

$$Precision_{micro-average}=\frac{Total\;true\;positives}{Total\;true\;positives+Total\;false\;positives}$$

$$Recall_{micro-average}=\frac{Total\;true\;positives}{Total\;true\;positives+Total\;false\;negatives}$$

$$F1_{micro-average}=\frac{Total\;true\;positives}{Total\;true\;positives+\frac{1}{2}\cdot \left(False\;positives+False\;negatives\right)}$$

Macro-averaging generalizes classification metrics, in a way that each class is given an equal weight. A macro-average metric is simply equal to the average of the metrics calculated independently for each class:

$$Metric_{macro-average\;}=\frac{1}{M}\overset{M}{\underset{i=1}{\sum }}metric_{i}$$

where M is the number of classes.

By a simple overview of the above formula, it is clear that the quality of the predictions for each class, regardless of the class prevalence in the dataset, has the same impact on the value of the macro-average metric. In that sense, macro-averaging is complementary to micro-averaging: it is useful when all classes, regardless of their size, are considered important and less useful when the focus is on the ensemble performance of the model, which is mainly determined by highly populated classes.

#### 1.4.2. Binary Segmentation Metrics

In the context of evaluating binary classification models, we employed several metrics that captured a different notion of similarity between prediction and ground-truth binary vectors. Both of these vectors have a length equal to the dataset size and each of their elements contain a label value (predicted or ground truth) corresponding to a particular dataset sample. By considering an image as the “dataset” and its pixels as “samples”, then, from a mathematical perspective, the output of a segmenter (predictor and ground truth) has no different form than of a classifier: it is a vector of zeros and ones, where each “1” corresponds to an “object” pixel and each “0” to a “background” pixel. Therefore, although they often come with different names (F1 score as Dice coefficient, accuracy as pixel accuracy, Jaccard index as intersection over union), all binary classification metrics are directly applicable within an image segmentation context, carrying the same advantages and drawbacks.

Although semantic segmentation can be reformulated as pixel classification, the transformation of the evaluation process into comparing flattened vectors leads to the collapse of the segmented image’s spatial structure. As a result, any idea of similarity beyond ensemble pixel matching cannot be investigated. For that reason, there exist certain metrics that are specialized for the evaluation of image segmentation models. The combined use of both types of metrics can provide an adequately thorough insight into the performance of the model.

#### 1.4.3. Extension of Binary Segmentation Metrics to Multiclass Problems

As in the classification case, binary segmentation metrics can be extended to multiclass problems by considering their micro- (possible for pixel classification metrics) and macro-averages.

## 2. How Good Is DL for Image Analysis in Esophageal Neoplasia?

### 2.1. Detection of Barrett’s Esophagus

According to the 2022 guidelines of the American College of Gastroenterology and the 2023 guidelines of the European Society of Gastrointestinal Endoscopy, Barrett’s esophagus (BE) is diagnosed in the presence of at least 1 cm in length of columnar epithelium containing intestinal metaplasia in the distal esophagus [58,59]. High-definition white-light endoscopy (HDWLE) and histopathological confirmation remain the gold standard for definitive diagnosis [58], while optical chromoendoscopy techniques are also employed with comparable outcomes. Although the use of CAD for the detection of BE may appear mundane, CAD could effectively decrease BE overdiagnosis and unnecessary surveillance. In patients diagnosed with BE after endoscopy, diagnosis was not histopathologically confirmed in 32.3% to 42% [60,61,62]. 

Pan et al. developed a DL algorithm in a neural network structure of fully convolutional networks (FCNs) to detect BE in endoscopic images via segmentation for the gastroesophageal (GEJ) and squamocolumnar junction (SCJ) [11]. Two different approaches were evaluated; in the first case, two independent FCNs were employed to separately segment either the GEJ or SCJ whereas in the second case, segmentation was achieved by a single network. The intersection over union (IOU, Jaccard index) was used as the performance metric. The separate segmentation approach yielded an average IOU of 0.56 for the GEJ and 0.82 for the SCJ, while the average IOU for the singe FCN was 0.66. Although the reported IOU for the SCJ is considered a satisfying score, a core limitation of this algorithm is that the length of the cephalad displacement of the SCJ is not taken into consideration in order to fulfill the endoscopic criterion for BE. This could limit potential prospective application for primary BE detection as it could lead to overdiagnosis.

Tsai et al. developed a DL model to detect BE in narrow-band imaging (NBI) endoscopy [12]. Prediction outcomes among six different pretrained models were evaluated and EfficientNetV2B2 [63] was selected, based on its superior accuracy. Upon training and validation, the CADe system yielded an accuracy of 94.37%, a sensitivity of 94.29% and a specificity of 94.44%.

Wu et al. proposed the Esophageal Lesion Network (ELNet) for the automatic classification and segmentation of esophageal lesions using CNNs [13]. This approach comprises four interdependent modules, namely a preprocessing module, a location module, a classification module and a segmentation module. The preprocessing module includes specular reflection removal to establish illumination uniformity, image normalization for dimension standardization and computational complexity reduction and data augmentation to alleviate overfitting. The location module utilizes Fast and Faster region-based CNNs (RCNNs) to locate regions of interest, which are subsequently clipped as image patches and function as the input for the classification module. The classification module employs a dual-stream network (DSN) that includes the Global Stream, which extracts global characteristics from inputting holistic images, and the Local Stream, which extracts local lesion characteristics from image patches, to categorize images as normal, inflammation, BE or cancer. The segmentation module employs three U-net segmentation networks [64] to perform segmentation specific to each lesion type, as classified by the classification module. This lesion-specific segmentation strategy was termed Segmentation Network with Classification (SNC). The ELNet exhibited notable performance with a classification and segmentation sensitivity of 90.3% and 80.1%, specificity of 90.3% and 96.5% and accuracy of 96.2% and 94.6%, respectively. Importantly, the DSN and SNC both outperformed several published state-of-the-art methods for classification and segmentation, respectively, in all three metrics.

Ali et al. described a DL system for the automatic identification, delineation and quantification of BE length and area for the accurate extraction of the Prague circumferential and maximum (C&M) extent scores [14]. The authors presented a DL-based depth estimator network that predicts the endoscopic camera distance from the gastric folds and automatically constructs depth maps, which allow for conversion from pixels to millimeters and, consequently, for the three-dimensional reconstruction of the esophageal surface. Segmentation of the GEJ and the BE area allows for C&M score calculation. This DL system was trained on three-dimensional-printed esophagus phantoms with varying BE patterns printed inside and was subsequently tested on 194 videos of HDWLE and NBI from 131 patients with BE that had previously received C&M scores from expert endoscopists. Across the entire patient cohort, the overall relative error was 8% and 7% and the mean difference was 3.6 mm and 2.8 mm compared to expert endoscopists’ measurements for C and M scores, respectively.

Bouzid et al. published a weakly supervised DL model based on multiple-instance learning (BE-TransMIL) for the detection of intestinal metaplasia in hematoxylin and eosin- or trefoil factor 3 (TFF3)-stained slides from capsule sponge samples [15]. Among the different encoders evaluated, ResNet50 [65] emerged as the best performing. For the H&E model, ResNet50 achieved a sensitivity of 72.7%, a specificity of 92.2%, an accuracy of 84.7% and an area under the receiver operating characteristic curve (AUROC) of 91.4%. For the TFF3 model, ResNet50 achieved an AUROC of 93.9%, an accuracy of 89.9%, a sensitivity of 79.1% and a specificity of 96.%. H&E BE-TransMIL was subsequently tested on an external dataset of 725 cases derived from the BEST2 trial [66] and achieved a comparable predictive performance with an AUROC of 87.3%, indicating an adequate generalizability.

### 2.2. Detection of Early Barrett’s Neoplasia

Upon establishment of BE diagnosis, regular endoscopic surveillance with four-quadrant biopsy sampling at one-to-two-centimeter intervals, per the “Seattle Protocol”, is recommended to detect dysplasia. Adherence to guideline recommendations remains a challenging endeavor as compliance rates greatly range from 51.2% to 86.8% [67,68,69], with an inverse correlation to the extent of BE diminishing to only 27% [69] and 30% [68] in patients with long and very long segment BE. Furthermore, the four-quadrant biopsy technique samples only 4–6% of the BE area, leading to significant biopsy sampling error [70]. These inherent limitations of current surveillance strategies lead to an unacceptably high rate of missed high-grade dysplasia and esophageal adenocarcinoma (19% and 24%, respectively [71]) that necessitate more efficient surveillance strategies.

De Groof et al. presented a hybrid ResNet/U-net model that achieved higher accuracy than endoscopists for the detection of neoplasia in HDWLE images of patients with BE [16]. Although ResNet models are generally used for image classification and U-net models for image segmentation, upon separate training, this approach involved joint training through multitask learning, allowing binary classification and subsequent neoplastic area delineation, if applicable. Five datasets were used for stepwise training, validation and testing. Pretraining involved the GastroNet dataset comprising 494,364 labeled GI endoscopic images, followed by refinement training with 557 NDBE images and 690 early neoplasia images from 414 BE patients. The DL system was validated internally using a set of 297 images with early neoplasia or NDBE achieving a high classification performance with an accuracy, sensitivity and specificity of 88%, 88% and 89%, respectively. In neoplastic images, the delineation score was 83% for the area that the majority of experts indicated as neoplastic. External validation was performed using two datasets of 80 images, each containing 40 NBDE and 40 neoplastic images. The DL system classified images with 89% accuracy, 90% sensitivity and 88% specificity in dataset 4. Dataset 5 was also evaluated by 53 international endoscopists; the DL system performed better in all primary outcome metrics, achieving a classification accuracy of 88% versus 73%, sensitivity of 93% versus 72% and specificity of 83% versus 74%, respectively. The overall delineation score was 93% versus 71%. The authors subsequently evaluated in vivo the diagnostic accuracy of their CAD system during live endoscopy in 10 patients with NDBE and 10 patients with BE neoplasia [72]. In a per patient analysis, the CAD system correctly identified 9 out of 10 patients designated as potentially neoplastic by the endoscopists; importantly, the single patient that was not detected by the CAD system did not show dysplasia upon histologic evaluation.

The same research group subsequently tested their custom-made hybrid ResNet/U-net model for BE neoplasia detection using NBI zoom images [17]. Using the same databases as described in their previous study for pretraining and refinement training, the researchers subsequently trained and internally validated their CAD system in a third dataset comprising 112 neoplastic and 71 NDBE NBI zoom images. A fourth dataset of 59 neoplastic and 98 NDBE NBI zoom videos was used for external validation. In order to compare the incremental benefit of different pretraining approaches, the same NBI videos were analyzed by the same DL model after pretraining with ImageNet or without pretraining. Regarding the performance on NBI zoom images, the proposed CAD system achieved an accuracy of 84%, a sensitivity of 88% and a specificity of 78% for the accurate discrimination of neoplastic BE and NDBE. In a per video analysis, the CAD system pretrained with endoscopic images achieved a better performance compared to no pretraining and ImageNet pretraining (accuracy of 83% versus 75% and 82%, respectively).

Fockens et al. developed a DL model for the detection of early neoplasia in BE that performed comparably to BE experts and more accurately than endoscopists, while it also increased their sensitivity for neoplasia detection when used as an assistive tool [18]. The proposed model was built by utilizing an EfficientNet-Lite1 encoder and a MobileNetV2 DeepLabV3+ decoder [73]. The DL model underwent initial pretraining with ImageNet followed by domain-specific pretraining using GastroNet. In the benchmarking image test set, the DL model achieved a classification sensitivity greater than 112 general endoscopists and comparable to 28 BE experts (90% versus 74% versus 87, respectively), while being inferior in terms of specificity. When assisting general endoscopists, the CADe system increased their sensitivity from 74% to 88%, without compromising the specificity. A similar performance pattern was observed in the benchmarking video test set. The authors subsequently tested their CADe system during the live endoscopic exploration of 15 neoplastic and 15 NDBE patients [74]. The CADe system accurately identified all neoplastic lesions, while incorrectly predicting neoplasia in eight patients (sensitivity of 100% and specificity of 47%).

Hussein et al. developed two DL models for the detection and localization of early neoplasia in BE [19]. The authors trained a model with a ResNet101 architecture [65] to classify images as either dysplastic or non-dysplastic, utilizing arbitrarily chosen frames from annotated videos of 118 patients. Subsequently, the authors trained a model with an FCNResNet50 architecture [75] to classify pixels into dysplastic or non-dysplastic using 192 images from 64 patients. The classification model achieved an AUC of 93%, a sensitivity of 91% and a specificity of 79% in the per image classification, while it correctly predicted dysplasia in 89.3% of patients. The segmentation model localized dysplasia with a sensitivity of 97%. Importantly, the DL model outperformed a panel of six non-expert endoscopists in dysplasia detection on a subset of testing set images (sensitivity and specificity of 96% and 88% versus 79% and 49%, respectively).

In a similar study, Abdelrahim et al. developed a hybrid CADe system consisting of a classification model based on the deep CNN, VSG16, and a segmentation model based on SegNet, a deep fully CNN architecture for semantic pixel-wise segmentation [76]. The training set comprised 75,198 images and videos of neoplastic BE from 96 patients and 1,014,973 images and videos from 65 patients with NDBE. In the image-based validation set, the DL model achieved a sensitivity, specificity and accuracy of 95.3%, 94.5% and 94.7%, respectively. A similar performance was achieved in the video-based validation set, where the CADe system significantly outperformed endoscopists in terms of sensitivity (93.8% versus 63.5%, *p* < 0.001), specificity (90.7% versus 77.9%, *p* < 0.028) and accuracy (92% versus 71.8%, *p* < 0.001) [20].

Hashimoto et al. described a DL model using CNNs to detect early neoplasia in endoscopic images from patients with BE [21]. The proposed CNN algorithm was pretrained on ImageNet and subsequently evaluated on a two-step task: correct binary classification of a given image as dysplastic BE or NDBE and accurate localization of the dysplastic region. Regarding binary classification, the sensitivity, specificity and accuracy were 96.4%, 94.2% and 95.4%, respectively. The mean average precision and IOU for the localization of the dysplastic region in positive images were 0.7533 and 0.3, respectively.

Ebigbo et al. developed a CAD system based on a CNN with a ResNet100 architecture for the diagnosis of early adenocarcinoma in patients with BE [22]. The proposed CAD system diagnosed neoplasia with a sensitivity of 97% and specificity of 88% for HDWLE images, outperforming 13 endoscopists (sensitivity of 76% and specificity of 80%). The DL model achieved a similar performance when given NBI images (sensitivity of 94% and specificity of 80%). The authors subsequently modified their DL model, adapting a state-of-the-art encoder–decoder network, and implemented it in real time during the endoscopic assessment of patients with BE, achieving a sensitivity of 83.7%, a specificity of 100% and an accuracy of 89.9% in 14 patients [77]. Struyvenberg et al. developed a CAD system using an ensemble of three deep CNNs based on the VGG16 architecture [78] for the detection of neoplasia in BE using volumetric laser endomicroscopy (VLE) [23]. Each of these networks were pretrained with the ImageNet database and were subsequently trained on a set of 172 VLE images from 22 patients. The CAD algorithm detected neoplasia with high accuracy and outperformed all 10 VLE experts (accuracy, sensitivity and specificity of 92%, 95%, 92% versus 77%, 70% and 81%, respectively). Fonolla et al. conducted a similar, multicenter study on the performance of an ensemble of DCNNs in the detection of neoplasia in patients with BE and reported an AUC of 0.96 [24].

Faghani et al. presented a DL model for the histologic diagnosis and grading of dysplasia in BE, using ResNet100, pretrained on the ImageNet database [25]. A total of 8596 histological images were obtained from 164 patients with NDBE, 226 with low-grade dysplasia (LGD) and 152 with high-grade dysplasia (HGD) and were allocated to a training, validation and test set with a 70:20:10 split. Overall, the presented model achieved a sensitivity and specificity of 81.3% and 100% for LGD and >90% for NDBE and HGD.

### 2.3. Esophageal Squamous Cell Carcinoma Detection

The application of DL methods for the endoscopic diagnosis of esophageal cancer, particularly esophageal squamous cell carcinoma (ESCC), has been extensively studied in a plethora of published studies. In a meta-analysis of 28 studies evaluating DL models for the diagnosis of early BE neoplasia or ESCC, the pooled accuracy, sensitivity, specificity, PPV, NPV and AUROC were 92.9%, 93.8%, 91.73%, 93.62%, 91.97% and 0.96, respectively [79].

Gong et al. developed a DL model for the endoscopic diagnosis of ESCC using HDWLE, which, upon satisfying internal test performance (accuracy of 95.6%, precision of 78%, recall of 93.89% and F1 score of 85.2%), underwent prospective multicenter external testing [26]. In five external test sets involving a total of 836 images, the established model achieved an accuracy of 93.9%, an average precision of 77.7%, an average recall of 72.5% and an average F1 score of 75%. Importantly, there was no statistically significant difference in the number of correctly identified regions of interest between the DL model and an expert endoscopist.

In a similar study, Liu et al. constructed a DCNN-based model for ESCC detection and margin delineation using HDWLE images [27]. The established model achieved an accuracy, sensitivity, specificity, PPV and NPV of 84.5%, 89.5%, 79.0%, 82.6% and 87.7% in the external validation set, while it achieved a superior performance compared to senior endoscopists and comparable to expert endoscopists in delineating ESCC margins (accuracy of 98.1% versus 78.6% and 95.3%, IoU of 76.2% versus 60.5% and 64%, respectively).

Τang et al. reported a real-time DCNN system that demonstrated excellent performance in diagnosing ESCC in the external validation sets and was also superior to expert endoscopists in terms of accuracy (91.3% versus 87.1%, *p* < 0.001), sensitivity (97.9% versus 85.0%, *p* < 0.001) and NPV (99.1% versus 93.5%, *p* < 0.001) [29].

Cai et al. developed another DCNN system for the diagnosis of early ESCC that proved better than junior, mid-level and senior endoscopists in terms of sensitivity (97.8% versus 83.0, 92.3 and 94.0%) and NPV (97.6% versus 85.6, 92.7 and 94.3%), while it was inferior in terms of PPV and specificity compared to the all endoscopists group [32]. Importantly, all performance metrics of the participating endoscopists were significantly improved upon utilization of the CAD system.

Fukuda et al. developed a CAD system based on the VGG16 architecture for the real-time diagnosis of ESCC using images of various endoscopic modalities [33]. The established DL system performed significantly better compared to expert endoscopists, yielding a sensitivity of 86% versus 74% (*p* < 0.01), specificity of 89% versus 76% (*p* < 0.01) and accuracy of 88% versus 75% (*p* < 0.01).

Yuan et al. developed a DL model based on the YOLOv3 architecture for ESCC detection in HDWLE and NBI images that emerged as more sensitive compared to 11 experienced endoscopists (90.8% versus 82.5%, *p* = 0.022) [28]. The constructed model also exhibited a satisfying performance in videos with a sensitivity of 89.5–100% and a specificity of 73.7% and 89.5%.

Wang et al. developed a single-shot multibox detector (SSD) using a CNN for the diagnosis of low-grade and high-grade squamous dysplasia and ESCC [34]. In this single-center, retrospective study, the established DL model achieved a satisfying diagnostic performance with an accuracy, sensitivity, specificity, PPV and NPV of 96.2%, 70.4%, 92.7%, 82.6% and 92.0%, respectively. Interestingly, the diagnostic accuracy of the SSD was higher in NBI images than WLI images (95% versus 89%) and in ESCC compared to high- and low-grade dysplasia (sensitivity of 98.9% versus 87.2% and 83.4%).

Li et al. conducted a multicenter, retrospective comparative study using a DCNN-based model for ESCC detection using NBI or WLI and concluded that CAD-NBI exhibited a superior accuracy (94.3% versus 89.5%, *p* = 0.028) and specificity (96.7% versus 83.1%, *p* < 0.001) and inferior sensitivity (91.0% versus 98.5%, *p* = 0.006) compared to CAD-WLI [35]. Furthermore, when compared to 20 endoscopists of varying experience, CAD-NBI outperformed mid-level and junior endoscopists in terms of all employed evaluation metrics, while it achieved a similar performance compared to experienced clinicians (accuracy of 94.3% versus 93.6%, respectively). The CAD-WLI also outperformed non-experienced endoscopists in all employed metrics and experienced endoscopists in sensitivity (98.5% versus 94.7%). Importantly, all three groups of endoscopists improved their diagnostic performance with the assistance of either CAD-WLI or CAD-NBI, although the increase was not statistically significant in the expert group.

Everson et al. developed a CADe system using a reformulated ResNet-18 model for the prediction of early ESCC based on an intrapapillary capillary loop (IPCL) pattern. The DL model demonstrated a similar real-time diagnostic performance compared to international expert endoscopists, achieving an average accuracy, sensitivity, specificity and F1 score of 91.7%, 93.7%, 92.4% and 94% versus 94.7%, 97%, 88% and 96.5%, respectively [30]. In a similar, single-center prospective study, Zhao et al. evaluated a DL model employing the VGG16 architecture for the diagnosis of early ESCC using NBI magnifying endoscopy [31]. The diagnostic performance of the established model (89.2%) was comparable to that of the senior endoscopists group (average accuracy of 92%) and superior to that of the mid-level and junior groups (82.0% and 73.3%, respectively).

### 2.4. Segmentation of Gross Tumor Volume

Jin et al. developed a mixed 3D V-Net [80] and 2D U-net [64] architecture (VUMix-Net) for the accurate automated delineation of esophageal gross tumor volume (GTV) using computer tomography (CT) scans [44]. A total of 185 CT scans were used in the training–validation cohort and 30 CT scans were included in the test cohort. VUMix-Net outperformed V-Net and U-Net in terms of the 2D and 3D Dice similarity coefficient (DSC), achieving a mean 2D-DSC of 68% and a mean 3D-DSC of 86%. Importantly, 99.5% to 99.8% of GTVs outlined by VUMix-Net were evaluated as clinically acceptable by two expert radiation oncologists.

Cao et al. proposed a deep dilated convolutional U-net model for the automated delineation of the clinical target volume (CTV) of esophageal cancer after esophagectomy [45]. Dilated convolution facilitates an expanded receptive field without a concurrent increase in the number of parameters, via kernel expansion through the introduction of holes between its successive elements [81]. A total of 3482 CT slices from 72 patients were used in the training and validation set with an 80:20 split, while the test set comprised 1104 CT slices from 19 patients. The proposed model segmented the CTV with a mean DSC of 86.7% and a respective 95% Hausdorff distance of 37.4 mm, outperforming the U-Net [64] and attention U-Net [82] models.

Jin et al. introduced a progressive semantically nested network (PSNN) segmentation model for GTV and CTV contouring in esophageal cancer radiotherapy [46]. The authors designed this architecture via reversing the direction of deeply supervised pathways in the progressive holistically nested network (PHNN) [83] and combining it with U-Net. Tested in a dataset of 148 esophageal cancer patients with paired positron emission tomography (PET)/CT and radiotherapy CT images, the proposed model achieved a DSC of 79% for GTV segmentation and a DSC of 82.6% for CTV segmentation. Yue et al. utilized the same model for GTV delineation on a cohort with 164 PET/CT scans of patients with ESCC. The authors reported a DSC of 72%, a mean surface distance of 2.43 mm and a Hausdorff distance of 11.87 mm [47].

Ye et al. developed a two-streamed DL method for the automated segmentation of esophageal GTV, using pretreatment CT and PET/CT images, based on the PSNN model proposed by Jin et al. [46,48]. The study enrolled 606 patients from multiple institutions assigned into a training–validation cohort of 148 patients, an internal testing set of 104 subjects from a single institution and an external testing cohort of 354 patients from multiple centers. The proposed model exhibited good contouring accuracy in the external testing set, achieving a mean DSC, HD95 and average surface distance (ASD) of 80%, 11.8 mm and 2.8 mm, respectively. These results showed no significant difference compared with those during internal testing, indicating good generalizability. Expert radiation oncologists’ evaluation of the model’s predictions showed that 88% were clinically acceptable. Importantly, out of 20 testing cases, the proposed model demonstrated comparable performance to that of four radiation oncologists, as assessed by means of the DSC and ASD (mean DSC of 82% versus 82% and ASD of 2 mm versus 1.9 mm, respectively).

### 2.5. Pretreatment Prediction of Patient Response

Hörst et al. developed two DL models that accurately predict and confirm the treatment response of patients with GEJ adenocarcinoma to neoadjuvant chemotherapy based on pretherapy and on-therapy histopathologic whole-slide images (WSIs) [39]. Of the several evaluated DL algorithms, the two best-performing network combinations were the CLAM network pretrained on The Cancer Genome Atlas (TCGA) data for non-small-cell lung cancer in combination with ResNet50 with ImageNet-based network weights and the pan-cancer TCGA pretrained ViT-4096 encoder network in combination with mean pooling. Clustering-constrained-attention multiple-instance learning (CLAM) is a weakly supervised DL method for WSI processing and learning, necessitating solely slide-level labels [84]. A total of 152 WSIs from 42 patients scheduled to receive neoadjuvant chemotherapy were used and allocated into a training set with 90 WSIs and a validation set with 62 WSIs. A total of 204 WSIs from 62 patients 14 to 21 days (d14-21) after treatment completion were used and allocated into a training set with 147 WSIs and a validation set with 47 WSIs. Overall, the pretrained CLAM network with ResNet50 outperformed all other methods in pretherapy specimens for the prediction of response, achieving a validation AUROC of 0.81 and an area under the precision–recall curve (AUPRC) of 0.82. The combination of the ViT-4096 encoder and mean pooling was the best performing on the on-therapy specimens for the confirmation of response with an AUROC of 0.84 and an AUPRC of 0.82.

Xie et al. presented a DL radiomics model for radiotherapy response prediction in patients with advanced esophageal SCC based on baseline CT scan characteristics [40]. A total of 248 patients scheduled to receive radiotherapy underwent baseline and post-treatment CT. Two types of radiomics models were evaluated, ML- and DL-based; the DL radiomics model using ResNet50, pretrained on ImageNet, was the best-performing method in predicting response. The DL model achieved AUCs of 0.876, 0.802 and 0.732 in primary, internal and external cohorts versus 0.767 and 0.594 in the training and internal validation cohorts of the ML model.

In a similar study, Hu et al. developed prediction models based on DL or handcrafted radiomics methods for chemoradiotherapy treatment response prediction in patients with ESCC using baseline CT scans [38]. Six CNNs including Xception, VGG16, VGG19, ResNet50, InceptionV3 and InceptionResNetV2 were pretrained on the ImageNet database. Baseline CT scans from 231 patients were collected and assigned to a training cohort containing 161 patients and a testing cohort with 70 patients. Of the assessed models, the model using ResNet50 achieved a superior classification performance (AUC of 0.805, accuracy of 77.1%, sensitivity of 83.9% and specificity of 71.8%) compared to the handcrafted radiomics model and the other CNNs.

Li et al. developed a pretreatment CT-based three-dimensional DL radiomics model (3D-DLRM) for the prediction of chemoradiation response in patients with esophageal SCC [37]. The 3D-DLRM was constructed via modification of the 2D convolution kernel of ResNet34 to 3D, thus obtaining a 3D basic block. The study dataset comprised baseline chest CT scans from 306 patients, allocated to a training (*n* = 203) and a validation set (*n* = 103). Overall, the 3D-DLRM showed a notable prediction performance, achieving an AUC of 0.833, an accuracy of 95.2% and a positive predictive value (PPV) of 100% in the validation cohort. The 3D-DLRM was also accurate in predicting the treatment response according to different radiotherapy regimens.

Kawahara et al. proposed a DL model for pathological complete response prediction after neoadjuvant chemoradiotherapy in patients with ESCC based on baseline endoscopic images [41]. The authors tested their 16-layer CNN using different imaging filters, of which the wavelet filter significantly improved the prediction performance of the model. Overall, the proposed DL model achieved an accuracy of 81%, a sensitivity of 0.80, a specificity of 81% and an AUC of 0.83.

Wang et al. developed a DL radiomics (DLR) nomogram to predict 3-year OS after chemoradiotherapy for esophageal carcinoma based on pretreatment CT images [43]. The authors adopted the DenseNet-169 architecture [85] for DLR feature extraction. The DLR nomogram outperformed the handcrafted radiomics model, achieving an AUC of 0.942 versus 0.665 in the validation cohort, respectively.

Yang et al. built a 3D-CNN based on ResNet to predict the survival status of patients with ESCC after one year based on PET scans [36]. The authors generated several models by modifying the components of the baseline model; the 34-layer network performed better irrespective of pretraining and attained an AUC of 0.738 in identifying patients who died within one year of the diagnosis.

### 2.6. Post-Treatment Evaluation of Patient Response

Matsuda et al. developed a DL algorithm for the evaluation of endoscopic response after neoadjuvant chemotherapy in patients with ESCC [42]. For their model, the authors used a modified version of AlexNet [86]. Two endoscopic images from each of 193 patients were used for model training and validation with a ratio of 7.5:1. Subsequently, the model was externally validated using 20 new endoscopic images from 20 patients. A median sensitivity, specificity, PPV, negative predictive value (NPV) and accuracy of 60%, 100%, 100%, 71% and 80%, respectively, were achieved versus 80%, 80%, 81%, 81% and 82.5% achieved by four endoscopists.

### 2.7. Surgical Phase Recognition and Intraoperative Detection of Anatomical Structures

DL-mediated video data analysis is a promising approach in offering intraoperative cognitive assistance in minimally invasive surgery (MIS) [87]. Although still in its infancy, intraoperative video data analysis using DL has yielded positive preliminary results in several tasks, including instrument recognition, phase recognition, detection of key anatomical structures, action recognition and gauze detection [88].

Takeuchi et al. proposed a DL model using Temporal Convolutional Networks for the Operating room (TeCNO) [89] for automated surgical phase recognition during robot-assisted minimally invasive esophagectomy (RAMIE) [49]. A total of 31 videos from patients undergoing RAMIE performed by the same practitioner were collected and allocated into two groups, namely an early period group comprising the first 20 cases performed and a late group comprising the following 11 cases. The surgical procedure was classified into nine phases and annotation was performed manually by two surgeons. The true positive rates ranged from 58% to 93%, while the model’s overall accuracy was 84%. Subsequently, the researchers compared the phase duration between the early and late group and reported a significant association with surgical proficiency, indicating that their DL model could function as an automated system of surgical skill evaluation.

Sato et al. presented a DL model for the real-time intraoperative identification of the recurrent laryngeal nerve during thoracoscopic esophagectomy compared to specialized esophageal surgeons and general surgeons [50]. The authors used DeepLabv3+ [90], a deep convolutional neural network model that is highly accurate in multi-scale semantic segmentation, pretrained on the 2012 PASCAL Visual Object Classes Challenge dataset [91]. The Dice coefficient was used as the performance metric. The DL model outperformed general surgeons in terms of the average Dice coefficient (0.58 versus 0.47, *p* = 0.019) while it was inferior to expert surgeons (0.58 versus 0.62).

Den Boer et al. described a U-net model with an EfficientNet-b0 encoder [63] for the detection of key anatomical structures in RAMIE video frames [51]. Pretraining using either ImageNet [92] or GastroNet [93] or no pretraining was used. Pretraining with ImageNet resulted in a higher mean Dice coefficient for the segmentation of the vena cava, aorta and the lung (0.79, 0.74 and 0.89, respectively) compared to GastroNet or no pretraining. Notably, the accuracy of the DL model was superior to that of an expert surgeon for the vena cava (0.79 versus 0.7), comparable for the lung (0.89 versus 0.9) and inferior for the aorta (0.74 versus 0.88).

## 3. Limitations

DL models entail protracted training cycles and extensive high-quality data requirements to acquire precise representations and generate reliable predictions [94]. Essential requirements of high-quality datasets include good image quality, adequate data diversity and balanced data, large size, lack of bias and accurate annotation [95]. Manual annotation with ground truth is a time-consuming process that requires high domain expertise and cross-evaluation to minimize erroneous labeling. Furthermore, the acquisition of large sets of labeled medical images, particularly in highly specialized tasks such as minimally invasive operations, is often challenging; data scarcity comprises a major burden that hinders the development and restricts the generalizability of DL models [95]. Several techniques have been developed to compensate for the scarcity of annotated medical image datasets. Transfer learning comprises an extensively utilized concept that denotes the repurposing of an already trained model to address a new task [96]. Therefore, instead of initiating the learning process from scratch, transfer learning leverages knowledge gained from the source task to enhance the model’s performance on the task at hand. Self-supervised learning is another beneficial technique in alleviating extensive data and annotation requirements; in this technique, DL models are trained using the unlabeled portion of the input data, for which they auto-generate labels, to learn the rest of the input data, thus converting unsupervised to supervised learning [97]. Generation of synthetic datasets through image data augmentation techniques also compensates for insufficient training data. During such processes, image manipulation yields training data with similar features as the original input data, albeit perceived as alien data by the DL model, thus increasing the effective dataset volume [98]. Importantly, the exposure of the model to altered images during training is expected to increase the generalizability and robustness in a real context. Furthermore, the increasing number of publicly available large-scale annotated image datasets could be employed to enhance pretraining or training data, as leveraged in many of the studies discussed above, beyond the capacities of a single research group.

CAD studies are often subject to several types of bias that ultimately affect the DL model’s performance [99,100]. Selection bias emerges upon the non-random selection of data that results in a database that is not representative of the average-risk population. Most DL studies in endoscopy utilize datasets retrieved from expert centers that do not encompass the inherent variability observed in imaged lesions, as well as the variation in endoscopists’ expertise and imaging devices’ quality encountered in routine clinical practice. Moreover, such datasets are often enriched in diseased subjects and thus the predictive values obtained may not be applicable in non-expert centers with a lower disease prevalence. To this end, most publications were retrospective single-institution studies in expert, high-volume centers, using high-quality equipment and may not reflect real-world clinical settings, resulting in poor generalization.

The use of limited or too homogeneous training data can result in overfitting, namely memorization rather than learning. Overfitting leads to artificially inflated performance results that lack generalizability on new, unseen data. The risk of overfitting can be minimized with the use of large and heterogeneous image datasets along with videos, which contain an increased variety in image quality owing to artifacts, secretions and partly obscured lesions [101].

Furthermore, and perhaps most importantly, the inference process of such models is not always interpretable by humans. The representations that DL models learn may not be explainable and theoretical bounds on their performance may be non-existent, which could be a major issue in the case of an expert–model inference conflict. This black-box nature of most DL models constitutes a significant challenge towards successful implementation and integration into routine clinical practice. The need for interpretable models has increased the popularity of research in the field of explainable AI; however, especially in DL, we are still far from claiming the availability of such models [102].

## 4. Where Do We Stand in DL for Esophageal Cancer Image Analysis and Future Directions?

Despite the rapidly enriched literature, there are no approved DL models for routine clinical application by the FDA in any aspect of the diagnosis and management of esophageal cancer. The success of CADe systems for the real-time detection of colonic polyps in clinical trials and the subsequent approval in the US, Europe and Asia indicate the potential of DL models in upper GI endoscopy [103]. Currently published studies report consistently robust DL models that achieve a comparable performance or even outperform experts in diverse aspects of esophageal cancer management. A plethora of studies have proposed DL algorithms for the endoscopic evaluation of esophageal cancer and the delineation to CTV prior to radiotherapy, whereas more recent areas of research include the intraoperative surgical guidance, the prediction of response to systemic treatment and the evaluation of response after treatment. Albeit current data are promising, for DL models to become ready for routine clinical application, several technical hurdles should be addressed.

Although frequently overlooked, an in-depth understanding of the basic functional principles of deep learning and adequate familiarization with clearly defined, relevant technical terminology are essential for clinicians who engage with the use of DL models and the interpretation of their output. Bridging the communication gap between computer engineers and clinicians and allowing for clear interdisciplinary and doctor–machine communication [104] are essential for fostering an environment that will embrace the real-time, routine clinical application of DL models.

Furthermore, it is important to note that the stand-alone performance of a DL model does not equal clinical value, and great caution should be taken when interpreting results and extrapolating them to clinical settings. The presented studies have several limitations, as discussed in the previous section. Of particular note, they are mostly prospective, single-center studies, conducted in expert centers using high-quality equipment. These study settings render clinical applicability questionable given that they differ significantly from real-world clinical settings. When constructing a DL model for the primary detection of esophageal cancer in endoscopic or histopathological images, the model should be evaluated on input that is representative of the average-risk population, mimicking daily practice. If this is not the case, selection bias arises, and the model’s performance may not be replicated in non-expert centers with a lower prevalence of esophageal cancer.

Additionally, there exists an unmet need for large datasets of images relevant to the designated task that could be utilized for model pretraining. In the abovementioned studies, pretraining was performed using large, publicly available datasets of medical images such as ImageNet. In an interesting study, however, Struyvenberg et al. showed that their model performed better when pretrained with endoscopic images compared to ImageNet pretraining or no pretraining (accuracy of 83% versus 82% and 75%, respectively) [17]. Further studies are required to investigate this association.

Moreover, image analysis in endoscopy is particularly challenging given endoscopy in everyday clinical practice includes multimodal acquisition, varied views and mucosal alterations, resulting in highly variable images; the free-hand movement of endoscopists can cause additional challenges to the DL model. Well-curated, high-quality endoscopic images may fail to capture these variations and result in a significant drop in the model’s performance in everyday clinical applications. To this end, Srivastava et al. reported that most architectures suffered a drop in their performance of over 20% when different datasets were used for testing [105]. This is a highly interesting area of research in the field, given that most studies perform training, validation and testing using images from the same dataset; only 10 out of the 41 discussed studies performed external validation. Extensive evaluation of promising DL models in generalizability studies with external datasets is required to develop algorithms that will be adaptable to input derived from different clinical settings and diverse populations.

Perhaps the biggest challenge for DL models, particularly in endoscopy and surgery, is their implementation during real-time procedures. However, existing data are scarce. Although DL algorithms may appear robust in an ex vivo setting, real-time evaluation should be considered imperative to enable the accurate estimation of their clinical performance. Given that CAD systems are generally considered “low risk”, insufficient evaluation prior to their approval will lead to an abundance of commercialized algorithms that will merely add to the care of patients. International, multicenter, randomized controlled studies comparing standard procedures versus AI-assisted implementation in carefully curated benchmarking datasets and in real time should be employed to identify the DL models and the aspects of clinical practice where they are truly useful. These datasets should be characterized by sufficient heterogeneity, reflecting natural lesion appearance variability, adequate sample size and should be designed only for external validation and not for the training of candidate models.

Lastly, DL algorithms could also function as assessors of endoscopic quality or surgical proficiency; the latter has been published by Takeuchi et al. during RAMIE. In surgical operations, DL algorithms could function as an automated system of surgical skill evaluation via the assessment of operative phase duration. In endoscopy, given that DL models can only analyze what is shown to them, they could serve as quality controllers via assessing intraprocedural quality indicators including procedural time and adherence to biopsy protocols [106].

## 5. Conclusions

Deep learning models show promise in facilitating the diagnosis and management of esophageal cancer through the accurate and efficient analysis of endoscopic images, demonstrating high sensitivity and specificity. The current literature is being rapidly enriched with image analysis studies for the endoscopic and histopathologic diagnosis of esophageal neoplasia, gross tumor delineation, treatment response and intraoperative guidance. Importantly, most models exhibit superior or similar performance compared to expert clinicians and significantly enhance their diagnostic accuracy when used as adjuncts. However, the performance of DL models may be influenced by factors such as the diversity and quality of the training dataset, the type of endoscopic imaging used and the presence of confounding factors like inflammation or other esophageal lesions. Further research is needed to validate the performance of deep learning models across different clinical settings and patient populations, as well as to assess their impact on clinical outcomes and workflow efficiency.

## Figures and Tables

**Table 1:** Summary of common evaluation metrics used in classification or segmentation tasks.

Metric	Task	Formula	Explanation	Limitations
Accuracy 0≤Accuracy≤1	Classification, segmentation	$\frac{TP+TN}{TP+TN+FP+FN}$	Measures the proportion of correctly classified instances out of the total instances, therefore serving as an empirical estimate of the misclassification probability.	May not be suitable for imbalanced datasets and applications where certain classes are of higher importance. Considering the first case, a model that is biased towards the majority class would still achieve a high accuracy, while in the second case, accuracy assigns the same cost to all false predictions and thus makes no distinguishes between classes.
Precision 0≤Precision≤1	Classification, segmentation	$\frac{TP}{TP+FP}$	Measures the proportion of true positive predictions out of all positive predictions made by the model. Used in applications where the primal focus is on minimizing false positives.	Sensitive to imbalanced datasets, especially when the positive class is significantly outnumbered. In that case, precision is a statistical estimate derived by very few samples and thus not reliable. If positive is the majority class, then precision may not provide sufficient information about the false negative rates.
Recall/Sensitivity 0≤Recall≤1	Classification, segmentation	$\frac{TP}{TP+FN}$	Measures the proportion of true positives that are correctly identified by the model. Used in applications where it is essential to capture all positive instances, thus minimizing false negatives.	For the same reasons as with precision, recall is sensitive to dataset imbalance and may not be sufficient on its own to evaluate the model’s overall performance.
F1 score 0≤F1≤1	Classification, segmentation	$2\cdot \frac{Precision\cdot Recall}{Precision+Recall}=$ $=\frac{TP}{TP+\frac{1}{2}\cdot \left(FP+FN\right)}$	Defined as the harmonic mean of precision and recall, therefore providing a balance between them. Since precision aims at minimizing false positives and recall at maximizing true positives, a balanced metric is considered robust to imbalanced datasets and useful in applications where both types of errors are important to consider.	Assigning equal weight to both precision and recall may not be suitable for certain applications where only one type of error is critical.
Specificity 0≤Specificity≤1	Classification, segmentation	$\frac{TN}{TN+FP}$	Measures the proportion of true negatives that are correctly predicted by the model. In that sense, it is a dual metric to sensitivity, being used in applications where it is crucial to capture all negative instances, thus minimizing false positives.	Similar to precision and sensitivity, specificity may not be very informative in problems with heavily imbalanced datasets. In the case where negative is the majority class, then a biased model may tend to overestimate specificity at the expense of low false negative rates (low sensitivity). On the other hand, if negative is the minority class and has very few samples in the dataset, then specificity may not be representative of the model’s performance in identifying true negatives under real conditions.
Jaccard Index $0\leq J_{index}\leq 1$	Classification, segmentation	$\frac{TP}{TP+FP+FN}$	Aims to capture all positive instances, while accounting for both false positives and false negatives. It is similar to F1 score, though it assigns a heavier penalty on false predictions.	Alike with F1, it assigns the same weight to false positives and negatives, which may not be suitable for certain applications.
Area under the ROC curve (AUC) 0≤AUC≤1	Classification, segmentation	There is no general formula, since the shape of the AUC may vary in different applications. Given the TP-FP rate pairs, though, it can be computed numerically.	The ROC curve is a graph of the $TP\;Rate=g\left(FP\;Rate\right)\;$, function, computed for different discriminative thresholds. Since a TP rate (sensitivity) of 1 and an FP rate (1-specificity) of 0 would constitute an optimal condition, we conclude that the larger the area under the ROC curve, the closer to optimal the model design.	Again, it may not be suitable for applications where sensitivity and specificity are not equally important. Moreover, the discriminative thresholds used to plot the ROC curve may be difficult to obtain.
Average Hausdorff distance (HD) Not bound by default. Optimal value is 0.	Segmentation	$d\left(S_{pr},S_{gt}\right)=\frac{1}{\left|S_{pr}\right|}\cdot \underset{u\in S_{pr}}{\sum }min_{v\in S_{gt}}\left|\left|u-v\right|\right|$ $AHD\left(S_{pr},S_{gt}\right)=max\left(d\left(S_{pr},S_{gt}\right),d(S_{gt},S_{pr}\right))$ $S_{pr}:Set\;of\;predicted\;object\;segmentation$ pixels $S_{gt}:Set\;of\;ground\;truth\;object\;segmentation$ pixels $\left|\left|\cdot \right|\right|:\;Norm\;defined\;in\;the\;pixel\;space.\;$ $Typically,\;it\;is\;the\;L_{2}\;\left(Euclidean\right)\;norm$	Measures the distance between two sets of points (here, predicted and ground-truth object segmentation), based on how close each point of set A is to set B and vice versa. Therefore, it potentially allows for evaluating the localization similarity of the two segmented objects.	By definition, every point of the predicted object segmentation contributes with an equal weight to the calculation of the Hausdorff distance. As a result, there is a slight sensitivity to possible outliers and isolated points in the predicted segmentation.

Abbreviations: TP: true positive; TN: true negative; FP: false positive; FN: false negative.

**Table 2:** Summary of studies evaluating DL models for image analysis of endoscopic and whole-slide images for the diagnosis of BE, neoplasia in BE and ESCC.

Study Goal	Modality	Model	Study Type	Pretraining	Patients	Images	Training Set	Validation	Testing	Comparison with Physician	External Validation	Performance	Year	Reference
Detection of BE	WLI, NBI	FCNN	Single-center, retrospective	NR	187	443	354	NR	89	No	No	IOU: 0.66	2021	[11]
	NBI	EfficientNetV2B2	Two-center, retrospective	Yes	724	1024	771	193	160	No	No	Accuracy: 94.3% Sensitivity: 94.29% Specificity: 94.44%	2023	[12]
	WLI images	Fast and Faster R-CNN, DSN, U-net	Single-center, retrospective	NR	748	1051	80%	10%	10%	No	No	Classification: Sensitivity: 90.3% Specificity: 90.3% Accuracy: 96.2 Segmentation Sensitivity: 80.1% Specificity: 96.5% Accuracy: 96.2%	2021	[13]
Detection, delineation and quantification of BE length and area	WLI videos and images	ResNeXt-101	Two-center, retrospective	ImageNet	131	871	736	135	194 videos	Yes	No	Overall relative error for C score: 8% Overall error for M score: 7%	2021	[14]
Detection of intestinal metaplasia	WSI	ResNet50	Single-center, retrospective	ImageNet	1141	1141	912		229	No	Yes	H&E model Sensitivity: 72.7% Specificity: 92.2% Accuracy: 84.7% AUROC: 91.4% TFF3 model Sensitivity: 79.1% Specificity: 96% Accuracy: 89.9% AUROC: 93.9%	2023	[15]
Detection of neoplasia in BE	WLI images	ResNet/U-Net	Multicenter, retrospective	GastroNet	669	1704	297		NR	Yes	Yes, double external validation	External Validation Accuracy: 89% Sensitivity: 90% Specificity: 88% Comparison with physician Accuracy: 88% vs. 73% Sensitivity: 93% vs. 72% Specificity: 83% vs. 74%	2020	[16]
Detection of neoplasia in BE	NBI	ResNet/U-Net	Multicenter, retrospective	GastroNet	514	1587	1430		NR	No	Yes	Accuracy: 84% Sensitivity: 88% Specificity: 78%	2021	[17]
Detection of neoplasia in BE	WLI, endoscopic videos	EfficientNet-Lite1/MobileNetV2DeepLabV3+	Multicenter, retrospective	ImageNet, GastroNet	3110	14,855 and 619 videos	13,846	200 and 180 videos	409 and 251 videos	Yes	No	Comparison with expert endoscopists Sensitivity: 90% vs. 87%	2023	[18]
Detection of neoplasia in BE	WLI and opTical chromoendoscopy images	ResNet101	Single-center, prospective	NR	118	174,553	148,936	25,161	456	Yes	No	AUC: 93% Sensitivity: 91% Specificity: 79%	2022	[19]
Localization of neoplasia in BE		FCNResNet50			64	192	94	12	86			Sensitivity: 97%		
Detection of neoplasia in BE	WLI images and videos	VSG16, SegNet	Multicenter, prospective	NR	270	1,090,642	1,090,171	471 images and 75 videos	NR	Yes	No	Comparison with endoscopists Sensitivity: 93.8% vs. 63.5% Specificity: 90.7% vs. 77.9% Accuracy: 92% vs. 71.8%	2023	[20]
Detection of neoplasia in BE	WLI, NBI	Inception-ResNet-v2	Single-center, retrospective	ImageNet	39	458	458		NR	No	No	Sensitivity: 96.4% Specificity: 94.2% Accuracy: 95.4% IoU: 0.3	2020	[21]
Detection of neoplasia in BE	WLI, NBI	ResNet100	Single-center, retrospective	NR	113	248	NR	NR	NR	Yes	No	Comparison with expert endoscopists Sensitivity: 97% vs. 76% Specificity: 88% vs. 80%	2018	[22]
Detection of neoplasia in BE	VLE	VGG16	Multicenter, prospective	Imagenet	47	318	172	146	NR	Yes	No	Comparison with expert physicians Accuracy: 92% vs. 77% Sensitivity: 95% vs. 70% Specificity:92% vs. 81%	2021	[23]
Detection of neoplasia in BE	VLE	DCNN	Multicenter, retrospective	NR	45	15,963	8772	NR	7191	No	No	AUC: 0.96	2019	[24]
Histologic diagnosis and grading of BE	WSI	ResNet100	Single-center, retrospective	ImageNet	542	8596	70%	20%	10%	No	No	Sensitivity: 81.3% Specificity: 100%	2022	[25]
ESCC diagnosis	WLI	Neuro-T	Multicenter, prospective	NR	NR	5162	4387	NR	775	Yes	Yes	Accuracy: 93.9% Precision: 77.7% Recall: 72.5% F1 score: 75%	2022	[26]
ESCC detection and margin delineation	WLI	DCNN	Single-center, retrospective	NR	1239	13,083	10,467	1479 images for ESCC detection and 1114 for delineation	NR	Yes	Yes	Comparison with senior and expert endoscopists Accuracy: 98.1% vs. 78.6% and 95.3% IoU: 76.2% vs. 60.5% and 64%	2022	[27]
ESCC detection	WLI, NBI	YOLO V3	Multicenter, retrospective	NR	2521	51,105	45,770	6075	NR	Yes	Yes	Comparison with expert endoscopists Sensitivity: 90.8% vs. 82.5%	2022	[28]
ESCC diagnosis	WLI	DCNN	Multicenter, retrospective	NR	1321	5035	4002	333	NR	Yes	Yes	Comparison with expert endoscopists Accuracy: 91.3% vs. 87.1% Sensitivity: 97.9% vs. 85.0% NPV: 99.1% vs. 93.5%	2021	[29]
ESCC diagnosis	Magnified NBI images	ResNet18	Two-center, prospective	NR	115	67,742	54,193	6774	6774	Yes	No	Comparison with expert endoscopists Accuracy: 91.7% vs. 94.7% Sensitivity: 93.7% vs. 97% Specificity: 92.4% vs. 88% F1 score: 94% vs. 96.5%	2021	[30]
ESCC diagnosis	Magnified NBI images	VGG16	Single-center, prospective	Imagenet	219	1350	NR	NR	NR	Yes	No	Comparison with expert endoscopists Accuracy: 89.2% vs. 92%	2018	[31]
ESCC diagnosis	NBI	DCNN	Single-center, prospective	NR	798	2615	2428	187	NR	Yes	No	Comparison with expert endoscopists Sensitivity: 97.8% vs. 94.0% NPV: 97.6% vs. 94.3%	2019	[32]
ESCC diagnosis	WLI, NBI, chromoendoscopy images	VGG16	Single-center, retrospective	NR	2096	28,333	23,746	94	NR	Yes	No	Comparison with expert endoscopists Sensitivity: 86% vs. 74% Specificity: 89% vs. 76% Accuracy: 88% vs. 75%	2020	[33]
ESCC diagnosis	WLI, NBI	SSD	Single-center, retrospective	NR	46	1200	936	NR	264	No	No	Accuracy: 96.2% Sensitivity: 70.4% Specificity: 92.7% PPV: 82.6% NPV: 92.0%	2021	[34]
ESCC diagnosis	WLI, NBI	DCNN	Multicenter, retrospective	NR	759	5051	4735	316	NR	Yes	No	Comparison with expert endoscopists CAD-NBI: Accuracy of 94.3% vs. 93.6% CAD-WLI: Sensitivity of 98.5% vs. 94.7%	2021	[35]

Abbreviations: WLI: white-light imaging; NBI: narrow-band imaging; EC: esophageal cancer; ESCC: esophageal squamous cell carcinoma; FCNN: fully convolutional neural network; NR: not reported; CNN: convolutional neural network; DCNN: deep convolutional neural network; DSN: dual-stream network; CT: computed tomography; PET: positron emission tomography; PSNN: progressive semantically nested network; WSI: whole-slide images.

**Table 3:** Summary of studies evaluating DL models for image analysis for the prediction of survival and response to systemic therapy and the evaluation of response after treatment in patients with esophageal cancer.

Study Goal	Modality	Model	Study Type	Pretraining	Patients	Images	Training Set	Validation	Testing	Comparison with Physician	External Validation	Performance	Year	Reference
ESCC survival	PET scans	ResNet	Single-center, retrospective	Yes	548	548	438	110	NR	No	No	AUC: 0.738	2019	[36]
Prediction of respose to chemoradiotherapy for ESCC	CT scans	3D-DLRM	Multicenter, prospective	NR	306	306	203	103	NR	No	No	AUC: 0.833 Accuracy: 95.2% PPV: 100%	2021	[37]
Prediction of response to chemoradiotherapy for ESCC	CT scans	ResNet50	Two-center, retrospective	NR	231	231	161	NR	70	No	No	AUC: 0.805 Accuracy: 77.1% Sensitivity: 83.9% Specificity: 71.8%	2021	[38]
Prediction of response to neoadjuvant chemotherapy	WSI	CLAM/ResNet50	Multicenter, retrospective	ImageNeT, TCGA	104	356	237	109	NR	No	No	AUROC: 0.81 AURPC: 0.82	2023	[39]
Prediction of response to radiotherapy for ESCC	CT scans	ResNet50	Multicenter, retrospective	ImageNet	248	248	154	45	49	No	Yes	AUC: 0.594 in the external validation cohort	2023	[40]
Evaluation of pathological CR after neoadjuvant chemoradiotherapy for ESCC	WLI	DCNN	Single-center, retrospective	NR	98	98	72	NR	26	No	No	Accuracy: 81% Sensitivity: 80% Specificity: 81% AUC: 0.83	2022	[41]
Evaluation of response to NAC	HD-WLE	AlexNet	Multicenter, retrospective	NR	193	386	338	48	NR	Yes	Yes	Comparison with endoscopists Sensitivity: 60% vs. 80% Specificity: 100% vs. 80% PPV: 100% vs. 81% NPV: 71% vs. 81% Accuracy: 80% vs. 82.5%	2023	[42]
EC survival after chemoradiotherapy	CT scans	DenseNet-169	Single-center, retrospective	ImageNet	154	154	116	38	NR	No	No	AUC: 0.942	2022	[43]

Abbreviations: WLI: white-light imaging; NBI: narrow-band imaging; EC: esophageal cancer; ESCC: esophageal squamous cell carcinoma; FCNN: fully convolutional neural network; NR: not reported; CNN: convolutional neural network; DCNN: deep convolutional neural network; DSN: dual-stream network; CT: computed tomography; PET: positron emission tomography; PSNN: progressive semantically nested network; WSI: whole-slide images; CR: complete response.

**Table 4:** Summary of studies evaluating DL models for image analysis for intraoperative surgical guidance and the delineation of target volume prior to radiotherapy.

Study Goal	Modality	Model	Study Type	Pretraining	Patients	Images	Training Set	Validation	Testing	Comparison with Physician	External Validation	Performance	Year	Reference
Delineation of esophageal GTV	CT scans	VUMix-Net	Single-center, retrospective	NR	215	215	185		30	Yes	No	2D-DSC: 68% 3D-DSC: 86%	2022	[44]
Delineation of esophageal CTV	CT scans	DDU-net	Single-center, retrospective	NR	91	4586	2786	696	1104	No	No	Mean DSC: 86.7% 95% Hausdorff distance: 37.4 mm	2021	[45]
Delineation of esophageal GTV and CTV	PET/CT scans	PSNN	Single-center, retrospective	NR	148	148	N/R			No	No	GTV DSC: 79% CTV DSC: 82.6%	2020	[46]
Delineation of esophageal GTV	PET/CT scans	PSNN	Single-center, retrospective	NR	164	164	NR			No	No	DSC: 72% mean surface distance of 2.43 mm Hausdorff distance of 11.87 mm	2022	[47]
Delineation of esophageal GTV	CT and PET/CT scans	PSNN	Multicenter, retrospective	NR	606	606	148		104	Yes	Yes	DSC: 80% Average surface distance: 2.8 mm 95% Hausdorff distance: 11.8 mm	2021	[48]
Surgical phase recognition during RAMIE	Intraoperative videos	TeCNO	Single-center, retrospective	NR	31	31	31	NR	31	No	No	True positive rate: 58–93% Accuracy:84%	2022	[49]
Recurrent laryngeal nerve during thoracoscopic esophagectomy	Intraoperative video frames	DeepLabv3+	Single-center, retrospective	PASCAL visual object classes challenge dataset	28	3040	2700	300	40	Yes	No	Comparison with expert surgeons: Dice coefficient: 0.58 vs. 0.62	2022	[50]
Anatomical structure detection during RAMIE	Intraoperative video frames	U-net/EfficientNet-b0	Single-center, retrospective	ImageNet, GastroNet	83	1050	850	N/R	200	Yes	No	Comparison with expert surgeon Accuracy for vena cava: 79% vs. 70% Accuracy for the lung: 89% vs. 90% Accuracy for the aorta: 74% vs. 88%	2023	[51]

Abbreviations: WLI: white-light imaging; NBI: narrow-band imaging; EC: esophageal cancer; ESCC: esophageal squamous cell carcinoma; FCNN: fully convolutional neural network; NR: not reported; CNN: convolutional neural network; DCNN: deep convolutional neural network; DSN: dual-stream network; CT: computed tomography; PET: positron emission tomography; PSNN: progressive semantically nested network; WSI: whole-slide images; RAMIE: robot-assisted minimally invasive esophagectomy; GTV: gross tumor volume; CTV: clinical target volume.

## Data Availability

Data supporting the recommendations of this article are included within the reference list. Please contact the corresponding author for any further data request.
